# Small-Bowel Lesions in Patients Taking Direct Oral Anticoagulants Detected Using Capsule Endoscopy

**DOI:** 10.1155/2020/7125642

**Published:** 2020-08-11

**Authors:** Minoru Yamaoka, Hiroyuki Imaeda, Naoki Hosoe, Kazuaki Yoneno, Ryu Kanno, Takashi Mitsufuji, Takahiro Sasaki, Yuji Akiyama, Hideki Ohgo, Yuichi Morohoshi, Takanori Kanai, Toshimasa Yamamoto, Toshihide Mimura, Haruhiko Ogata, Nobuo Araki, Keiji Yamamoto, Hidetomo Nakamoto

**Affiliations:** ^1^Department of General Internal Medicine, Saitama Medical University, Japan; ^2^Department of Gastroenterology, Saitama Medical University, Japan; ^3^Center for Diagnostic and Therapeutic Endoscopy, Keio University, Japan; ^4^Department of Gastroenterology, Saitama Medical Center, Japan; ^5^Department of Neurology, Saitama Medical University, Japan; ^6^Department of Rheumatology, Saitama Medical University, Japan; ^7^Department of Gastroenterology, Yokohama Municipal Citizen's Hospital, Japan; ^8^Division of Gastroenterology and Hepatology, Department of Internal Medicine, School of Medicine, Keio University, Japan; ^9^Department of Cardiology, Saitama Medical University, Japan

## Abstract

**Methods:**

This study was a prospective, open-label, nonblinded, multicenter, and observational study. From September 2013 to March 2017, patients taking DOACs were enrolled. Patients underwent VCE. The type and location of small-bowel lesions were registered. Also, (1) the proportion of lesions detected between types of DOAC was evaluated and (2) the hemoglobin (Hb) and serum ferritin levels were compared between patients with and without small-bowel lesions.

**Results:**

33 patients were enrolled, but 4 patients withdrew their consent, and VCE was performed on 29 patients. Eight, 13, and 8 patients received dabigatran, rivaroxaban, and apixaban, respectively. Small-bowel transit was complete in 27 of 29 patients (93.1%). Small-bowel lesions were detected in 23 (79.3%), redness in 12 (41.4%), erosions in 14 (48.3%), and angioectasia in 3 (10.3%) patients, and 6 patients (20.7%) had no abnormalities. Redness and erosions were detected in the upper, middle, or lower portions, but erosions tended to be less frequent in the middle portion (*p* = 0.25, 0.06). Angioectasia was not detected in the lower portion. No patients had active bleeding. The findings did not differ according to the drug. The relationships between the endoscopic findings and the Hb and serum ferritin levels were not significant.

**Conclusion:**

Many patients taking DOACs had small-bowel lesions; however, most lesions were relatively mild. Observing small-bowel lesions over longer periods may be necessary in patients taking DOACs. This trial is registered with UMIN000011527.

## 1. Introduction

Since Leung et al. reported a single case of low-dose aspirin- (LDA-) induced multiple small intestinal ulcers in 2007 [1], many investigators have described LDA-induced small intestinal mucosal injuries. Watanabe et al. reported that all of their patients who used LDA had small intestinal lesions that were detected using capsule endoscopy (CE) [2]. Iwamoto et al. investigated 181 patients who underwent CE for occult bleeding, and erosions were observed in 45 cases, 27 of whom were taking LDA or non-LDA nonsteroidal anti-inflammatory drugs [3]. Endo et al. described small intestinal lesions in 31% of 13 subjects who took aspirin (100 mg) for 2 weeks [4]. Shiotani et al. performed CE on 20 young healthy individuals before and after medium doses of enteric aspirin were administered for 100 days and rabeprazole (10 mg) was administered for 1 week and found large erosions that included 2 small intestinal ulcers in 60% of the subjects [5].

Recently, direct oral anticoagulants (DOACs) have been administered as alternatives to warfarin. Dabigatran is a direct thrombin inhibitor, and rivaroxaban, apixaban, and edoxaban are factor Xa inhibitors. DOACs have significantly fewer side effects than warfarin, including intracranial hemorrhage; hence, the number of patients taking DOACs is gradually increasing [6–8]. However, the findings from meta-analyses have shown that compared with warfarin, the incidence of gastrointestinal (GI) bleeding is higher in association with DOACs [9, 10]. The causes of GI bleeding in association with DOACs were bleeding from colon or gastric cancers and diverticular hemorrhages. Compared with warfarin, dabigatran and rivaroxaban are associated with higher risks of GI bleeding, depending on their doses, and the risk of GI bleeding associated with apixaban is comparable [11]. Edoxaban is associated with significantly less GI bleeding at low doses (30 mg once daily) compared with warfarin, but the risk is significantly greater at high doses (60 mg once daily) [10]. But unlike Western countries, the rate of GI bleeding for both dabigatran and rivaroxaban is equivalent to warfarin in Asian countries [12]. Furthermore, edoxaban tends to cause less digestive tract bleeding than warfarin [13]. Esophageal ulcers caused by dabigatran have been described by Toya et al. [14], Kasai et al. [15], and Okada and Okada [16]. The tartaric acid coating on dabigatran causes esophageal mucosal disorders, because dabigatran persists in the midesophagus if it is consumed without water [17]. However, there have been no reports of small intestinal lesions in patients who receive DOACs. Here, we aimed to evaluate small intestinal mucosal injuries in patients taking DOACs using video capsule endoscopy (VCE).

## 2. Methods

This study was a prospective, open-label, nonblinded, multicenter, and observational study. From September 2013 to March 2017, pat5ents taking DOACs, namely, dabigatran, rivaroxaban, and apixaban, for atrial fibrillation at Saitama Medical University Hospital, Keio University Hospital, Saitama Medical Center, and Yokohama Municipal Citizen's Hospital were enrolled. Patients with severe comorbidities, including severe anemia and exacerbations of heart failure that required blood transfusion, Crohn's disease, and ileus, were excluded.

**Figure 1:**
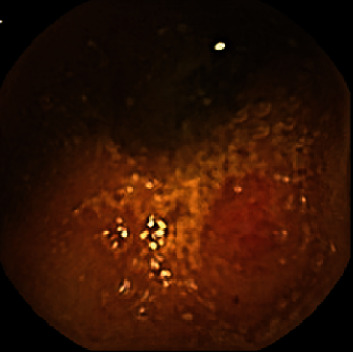
Redness.

**Figure 2:**
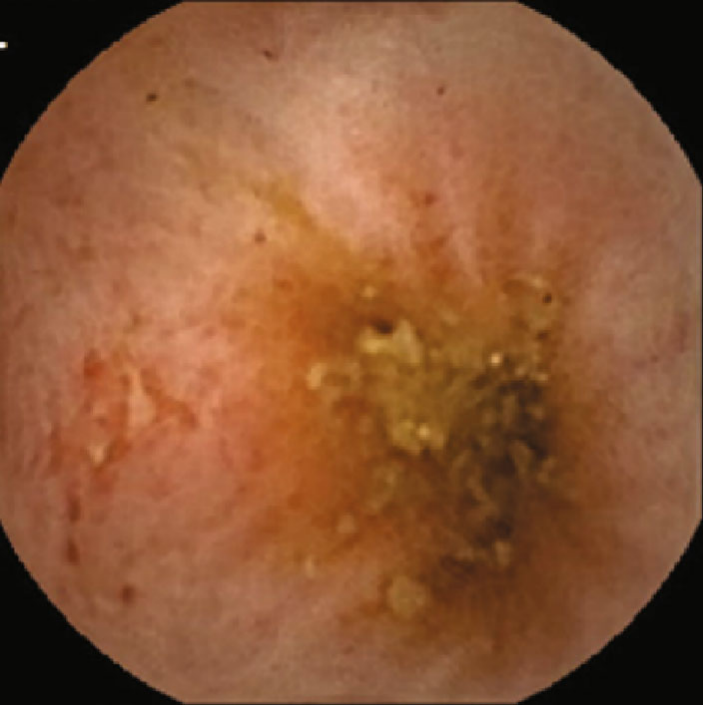
Erosion.

**Figure 3:**
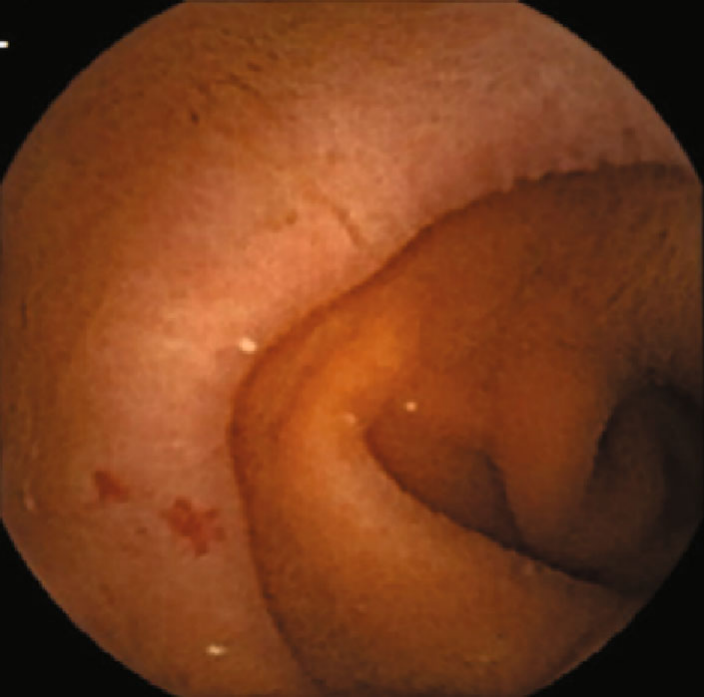
Angioectasia.

The hemoglobin (Hb) and serum ferritin levels, the esophagogastroduodenoscopy (EGD) findings, and colonoscopic findings were examined. VCE (PillCam SB2 Given Imaging Ltd., Yoqneam, Israel) was performed to examine small intestinal lesions according to the DOAC used. Redness, erosion, ulcer, and angioectasia were checked. Redness was a red spot (Figure 1), and erosions were defined as small and superficial mucosal disruptions denuded of villi (Figure 2). Ulcers were defined as large submucosal disruptions with a central area covered with exudate, and angioectasia was a patchy erythematous lesion (Figure 3). The images were analyzed using the proprietary RAPID 6.5 software by an expert (N. H.) who had performed more than 150 VCE examinations blindly. The type and location of small-bowel lesions were registered. Also, (1) the proportion of lesions detected between types of DOAC was evaluated and (2) the hemoglobin (Hb) and serum ferritin levels were compared between patients with and without small-bowel lesions.

The study protocol accorded with the tenets of the revised Declaration of Helsinki (1989), and it was approved by the institutional review boards at our institutions. Written informed consent was obtained from all of the patients. This study was registered with the University Hospital Medical Information Network Clinical Trials Registry (UMIN000011527, October 1, 2013).

IBM®SPSS® statistical software version 24 (IBM Corporation, Armonk, NY, USA) was used for the statistical analyses. The data were analyzed using *t*-tests and Fisher's exact test.

## 3. Results

Thirty-three patients were enrolled to participate in this study, but 4 patients withdrew their consent, and VCE was performed on 29 patients. The patients' mean age was 71 years (range 42–84 years), and 24 males and 5 females participated in this study. Twenty patients had atrial fibrillation, 7 had paroxysmal atrial fibrillation, 11 had hypertension, 7 had hyperlipidemia, and 6 had cerebral infarction. Eight patients took dabigatran, 13 took rivaroxaban, and 8 took apixaban. The mean duration of DOAC use was 10.4 months (2-34 months). Additionally, 1 patient took bayaspirin, 1 patient took celecoxib, 9 patients took proton pump inhibitors (PPIs), and 3 patients took H_2_ receptor antagonists. The average Hb concentration was 13.6 g/dL (range 8.8–16.5 g/dL) (Table 1). Twenty-two patients underwent EGD, and atrophic gastritis was present in 12 patients, hiatal hernias in 5 patients, gastric polyps in 5 patients, erosive gastritis in 3 patients, gastric ulcer or ulcer scar in 2 patients, reflux esophagitis in 2 patients, endoscopic submucosal dissection scar for early gastric cancer in 2 patients, and an esophageal ulcer in 1 patient. Nineteen patients underwent colonoscopy, and colonic polyps were present in 14 patients, colonic diverticulum were present in 4 patients, and a rectal ulcer was present in 1 patient. None of these lesions detected by EGD and colonoscopy had active bleeding.

The patients evaluated with VCE was 29. Redness in the lower esophagus was present in 1 patient, gastric erosions were present in 2 patients, and gastric redness was present in 1 patient (Table 2). The patient who had redness in the lower esophagus was taking apixaban. Small-bowel transit was complete in 27 of 29 patients (93.1%). Small-bowel lesions were observed in 23 of 29 patients (79.3%).

Redness was observed throughout the small intestine in 12 patients (41.4%), and it was present in the upper portion in 8 (27.6%), in the middle portion in 6 (20.7%), and in the lower portion in 7 (24.1%) patients (Table 2). Erosions were observed in 14 patients (48.3%), and they were present in the upper portion in 7 (24.1%), in the middle portion in 4 (13.8%), and in the lower portion in 10 (34.4%) patients (Table 2). Erosions tended to occur less frequently in the middle portion; however, the difference was not significant (*p* = 0.25 compared with the upper portion, *p* = 0.06 compared with the lower portion). Angioectasia was observed in 3 patients (10.3%) and was present in the upper portion in 2 patients (6.9%) and in the middle portion in 1 patient (3.4%) and was absent from the lower portion (Table 2). There were no ulcers in any patients. Erosions tended to be more frequent in patients taking dabigatran or apixaban than in patients taking rivaroxaban; this difference was not significant (*p* = 0.17). (Table 3). No significant differences were observed regarding angioectasia among the patients taking the different DOACs. None of these patients had active bleeding from small intestinal lesions.

The mean Hb concentrations in the patients with and without small-bowel lesions were 14 g/dL and 13 g/dL, respectively, a difference which was not significant (*p* = 0.38). The mean ferritin levels in the patients with and without small-bowel lesions were 82 mg/dL and 62 mg/dL, respectively, a difference which was not significant (*p* = 0.44).

## 4. Discussion

This study's findings showed that of the patients who took DOACs, 12 (41.4%) had redness, 14 (48.3%) had erosions or small ulcers, 3 (10.3%) had angioectasia, and 6 (20.7%) had no abnormalities in their small bowel. Small-bowel lesions were observed in 23 of 29 patients (79.3%); therefore, there was a high incidence of small-bowel lesions in patients taking DOACs. However, none of these patients had active bleeding, and most of the lesions were mild. Patients with severe anemia or active bleeding were excluded from this study; hence, only patients with mild symptoms were included. LDA-induced lesions cause redness, erosions, and ulcers [1–5]. Previous studies' findings that describe the characteristics of small-bowel injuries associated with chronic LDA use suggest that ulcers are observed mainly in the distal part of the small bowel [18–20]. In this study, erosions tended to be observed less frequently in the middle portion of the small bowel in the patients taking DOACs; however, there were no significant differences regarding the distributions of the lesions. There were no ulcers in any patients; therefore, the intake of DOAC might not be related with severe ulcers in the small intestine.

In this study, angioectasia was observed in the upper and middle portions, but not in the lower portion of the small bowel. Kaufman et al. used a transit time-based quartile method to evaluate 158 patients with angioectasia who underwent CE and found that most lesions (67.1%) were in the first quartile [21]. Igawa et al. reported that while there were no differences regarding the location of type 1a angioectasia among patients with occult gastrointestinal bleeding, type 1b angioectasia was relatively less frequent in the lower portion compared with that in the upper and middle portions of the small bowel [22]. The data reported before. Therefore, angioectasia might not be affected by the intake of DOACs. Comparison of VCE findings before and after the administration of D7-19ACs is needed.

No significant relationships were determined in relation to the presence of the Hb level or the serum ferritin level between the patients with and without small-bowel lesions in this study. Despite detecting abnormal findings in the small bowel, no active bleeding was seen by VCE and there was no severe anemia in any patients in this study. Furthermore, compared with warfarin, the incidence of GI bleeding is higher in association with DOACs. The causes of GI bleeding in association with DOACs are bleeding from colon or gastric cancers and diverticular hemorrhages; however, none of these lesions detected by EGD and colonoscopy had active bleeding. DOACs did not affect bleeding from the upper GI tract and the colon in this study.

No significant differences were observed among the DOACs in relation to small-bowel lesions. The findings from the Randomized Evaluation of Long-Term Anticoagulation Therapy trial of dabigatran showed that in the warfarin group, 5 patients with GI tract bleeding had gastric cancer or colonic cancer and that in the dabigatran group, 30 patients with GI tract bleeding had colonic cancer and 1 patient had gastric cancer [6]; hence, dabigatran might induce GI tract bleeding from colon cancer [17]. Rivaroxaban, apixaban, and edoxaban compete directly with the S1 pocket of factor Xa and inhibit factor Xa activity, whereas dabigatran is a prodrug that is activated in the presence of esterase in the GI tract, plasma, and liver. The causes of the mucosal damage by dabigatran were thought to be due to direct acting at the local area where it is absorbed. In addition, tartaric acid coats dabigatran tablets, and the tablets can cause mucosal damage if they are retained within the esophagus. While we expected an increase in the frequency of intestinal mucosal injury among the patients who took dabigatran as a consequence of the tartaric acid coating, this study's findings did not demonstrate a higher rate of small-bowel lesions associated with the use of this DOAC. The mechanisms underlying mucosal injuries caused by DOACs other than dabigatran remain unclear. Small-bowel lesions, including redness, erosions, and angioectasia, might be more easily detected by performing CE on patients who take DOACs, because the DOACs might cause bleeding that could facilitate the detection of the lesions.

This study has several limitations. While this was a multicenter study, the sample size was small. Hence, more patients should be accrued and investigated in the near future. Moreover, this study only included data that described the patients' findings after the administration of the DOACs, and data describing the findings before their administration were absent; therefore, it remains unclear whether small intestinal lesions are directly induced by DOACs. Studies of patients' findings before and after the administration of DOACs are needed in the near future. Furthermore, patients with severe anemia and overt bleeding were excluded from this study, and most of the enrolled patients did not have bleeding or had minor bleeding. Patients taking edoxaban were not included in this study, because edoxaban was not available in Japan when this study began.

## 5. Conclusion

Small-bowel lesions, including redness, erosions, small ulcers, and angioectasia, were detected in 23 of 29 patients who took DOACs. More patients using DOACs should be investigated using CE in the near future.

## Figures and Tables

**Table 1:** Patients' characteristics.

Parameter	
Mean age (years) (range)	71 years (42–84)
Sex, *n* (male/female)	24/5
Comorbid disease, *n*	
Atrial fibrillation	20
Paroxysmal atrial fibrillation	7
Hypertension	7
Hyperlipidemia	7
Cerebral infarction	6
DOAC, *n*	
Dabigatran	8
Rivaroxaban	13
Apixaban	8
Bayaspirin, *n*	1
Celecoxib, *n*	1
PPI, *n*	9
H_2_ blocker, *n*	3
Mean Hb (g/dL) (range)	13.6 (8.8–16.5)

DOAC: direct oral anticoagulant; PPI: proton pump inhibitor; Hb: hemoglobin.

**Table 2:** Capsule endoscopy findings.

Entire small intestine observation rate, *n* (%)		27/29 (93.1)

Detection rate, *n* (%)		23/29 (79.3)

Esophagus	Redness (lower), *n* (%)	1 (0.7)

Stomach	Erosions, *n* (%)	2 (6.9)
	Redness, *n* (%)	1 (0.7)

Upper small intestine	Redness, *n* (%)	8 (27.6)
	Erosions, *n* (%)	7 (24.1)
	Angioectasia, *n* (%)	2 (6.9)
	No abnormalities, *n* (%)	15 (51.7)

Middle small intestine	Redness, *n* (%)	6 (20.7)
	Erosions, *n* (%)	4 (13.8)
	Angioectasia, *n* (%)	1 (0.7)
	No abnormalities, *n* (%)	18 (62.1)

Lower small intestine	Redness, *n* (%)	7 (24.1)
	Erosions, *n* (%)	10 (34.5)
	Angioectasia, *n* (%)	0
	No abnormalities, *n* (%)	15 (51.7)

Entire small intestine	Redness, *n* (%)	12 (41.4)
	Erosions, *n*	14 (48.3)
	Angioectasia, *n*	3 (10.3)
	No abnormalities, *n*	6 (20.7)

**Table 3:** Findings at each small intestinal site according to the direct oral anticoagulant used.

	Dabigatran	Rivaroxaban	Apixaban
Site/finding	8	13	8
Upper small intestine			
Redness, *n*	3	3	2
Erosions, *n*	1	3	3
Angioectasia, *n*	1	0	1
Middle small intestine			
Redness, *n*	2	3	1
Erosions, *n*	2	1	1
Angioectasia	0	1	0
Lower small intestine			
Redness, *n*	2	1	4
Erosions, *n*	4	2	4
Angioectasia, *n*	0	0	1
Entire small intestine			
Redness, *n*	3	5	4
Erosions, *n*	5	4	5
Angioectasia, *n*	1	1	1

## Data Availability

This manuscript describes a study that was aimed at evaluating direct oral anticoagulant (DOAC) related to small-bowel lesions using video capsule endoscopy. We believe that our study makes a significant contribution to the literature, because its findings showed that many patients taking DOACs had small-bowel lesions; however, most lesions were relatively mild, and they did not cause bleeding.

## Abbreviations

DOAC: Direct oral anticoagulant
VCE: Video capsule endoscopy
LDA: Low-dose aspirin
CE: Capsule endoscopy
GI: Gastrointestinal
Hb: Hemoglobin
EGD: Esophagogastroduodenoscopy
PPI: Proton pump inhibitor.
